# Divergence of Mammalian Higher Order Chromatin Structure Is Associated with Developmental Loci

**DOI:** 10.1371/journal.pcbi.1003017

**Published:** 2013-04-04

**Authors:** Emily V. Chambers, Wendy A. Bickmore, Colin A. Semple

**Affiliations:** MRC Human Genetics Unit, MRC Institute of Genetics and Molecular Medicine, University of Edinburgh, Western General Hospital, Edinburgh, United Kingdom; University of Chicago, United States of America

## Abstract

Several recent studies have examined different aspects of mammalian higher order chromatin structure – replication timing, lamina association and Hi-C inter-locus interactions — and have suggested that most of these features of genome organisation are conserved over evolution. However, the extent of evolutionary divergence in higher order structure has not been rigorously measured across the mammalian genome, and until now little has been known about the characteristics of any divergent loci present. Here, we generate a dataset combining multiple measurements of chromatin structure and organisation over many embryonic cell types for both human and mouse that, for the first time, allows a comprehensive assessment of the extent of structural divergence between mammalian genomes. Comparison of orthologous regions confirms that all measurable facets of higher order structure are conserved between human and mouse, across the vast majority of the detectably orthologous genome. This broad similarity is observed in spite of many loci possessing cell type specific structures. However, we also identify hundreds of regions (from 100 Kb to 2.7 Mb in size) showing consistent evidence of divergence between these species, constituting at least 10% of the orthologous mammalian genome and encompassing many hundreds of human and mouse genes. These regions show unusual shifts in human GC content, are unevenly distributed across both genomes, and are enriched in human subtelomeric regions. Divergent regions are also relatively enriched for genes showing divergent expression patterns between human and mouse ES cells, implying these regions cause divergent regulation. Particular divergent loci are strikingly enriched in genes implicated in vertebrate development, suggesting important roles for structural divergence in the evolution of mammalian developmental programmes. These data suggest that, though relatively rare in the mammalian genome, divergence in higher order chromatin structure has played important roles during evolution.

## Author Summary

The mammalian genome is organised into large multi-megabase domains defined by their physical structure, or higher order chromatin structure. Although these structures are believed to be well conserved between species, there have been few studies attempting to quantify such conservation, or identify divergent structures. We find that regions showing clear evidence of divergence in higher order chromatin structure encompass at least 10% of the mammalian genome, and include many hundreds of genes whose regulation may have been affected. At least some of these genes have been directly implicated in evolutionary innovations to vertebrate developmental programmes, so divergent regions may have been disproportionately important during evolution. In addition, we show that divergent regions occur in large stretches of more than 2 Mb in the human genome and are enriched towards telomeres at the ends of human chromosomes. This may reflect shifts in the nuclear organisation and regulatory functions of chromatin domains between human and mouse.

## Introduction

Chromatin structure plays critical roles in genome functions such as transcription, replication and repair, it can mediate human disease processes [1] and is implicated in ageing [2]. The primary level of eukaryotic chromatin structure involves the DNA sequence wrapped around nucleosomes and the covalent modification of histones within the nucleosomes. Interactions between nucleosomes give rise to secondary structures, which may include a 30 nm chromatin fibre, and which vary in their degree of compaction across the genome [3]. Multiple higher levels of topological organisation, further structuring the genome, are also known to exist but their precise nature and their inter-relationships are the subjects of intense study and debate [4].

Genome-wide data relating to primary levels of chromatin structure (nucleosome occupancy, histone modifications etc) in a variety of mammalian cell types are abundant, due to the ability to profile these chromatin features by combinations of MNase digestion, chromatin immunoprecipitation and high-throughput sequencing [5]. However, the diversity of higher order structure across the genome is less well studied. An early genome-wide survey of higher order chromatin structure in the human genome discovered an undulating landscape of domains from hundreds of kilobases to many megabases in size; some relatively accessible or ‘open’ and others adopting a spectrum of more ‘closed’ condensed structures [3]. The most open domains corresponded to regions of relatively high gene density, replicating early in the cell cycle, and they may create an environment that facilitates transcriptional activation [6]. In contrast, more closed regions were relatively late replicating and gene poor. Replication timing profiles measured across the genome in multiple human and mouse cell types have also revealed the presence of domains on a similar scale, ranging from a few hundred kilobases to several megabases, that show coordinated replication timing during the cell cycle [7], [8]. Other studies have examined different facets of higher order chromatin structure and organisation. Genomic regions interacting with tagged nuclear lamina components, and hence considered to be located at the nuclear periphery, have been mapped across the human and mouse genomes [9], [10]. These lamina-associated domains (LADs) are relatively late replicating, gene poor regions from 40 Kb to 15 Mb in length and harbour genes with low transcriptional activity [10]. Overall LADs encompass around 40% of the genome and their locations and extent appear to be largely similar over cell types [10]. More recently, 3C-type physical contact maps, based on cross-linking frequencies, have been used to infer the spatial proximities and 3D- architecture between all possible 1 Mb segments of the human genome [11]–[13]. A familiar pattern of two spatial compartments within the nucleus also emerged from these data. One compartment composed of regions of gene rich, open, actively transcribed chromatin, and another containing regions with opposing features. These broad patterns emerge at the genome wide level, in spite of many regions that adopt cell type specific structures.

Remarkably, given the diverse methodologies used to investigate them, significant correlations have been found among some of these coarse-grained facets of higher-order genome organisation and function. There is a strong overlap between the sequences that replicate together during the same temporal window of S phase, and those sequences that can be captured together by Hi-C [12], [14], consistent with the idea that genomic regions in close proximity tend to replicate at similar times and thereby define important features of chromosome organisation. These may well equate to the replication foci visible in the nucleus [15]. It has long been known that globally late replication tends to occur at the nuclear periphery [16], [17] and this has been substantiated by more detailed analysis using fluorescence in situ hybridisation (FISH) of specific loci [7], [14]. There is also a correlation between late replicating chromosomal domains and LADs [10] but it is not absolute and the relationship tends to breakdown at LAD borders and at particular genes. Moreover, such correlations present a moving target as genomic patterns of replication timing domains and LADs change upon differentiation and re-programming [8], [10]. We also lack a comprehensive view of how genome-wide chromatin structure varies across cell types. Although cell type specific structures are clearly present, it seems that the higher order domains reflected in replication timing and Hi-C data remain largely unchanged over a variety of cell types and throughout the cell cycle [18], [19]. Key questions in chromatin structure and nuclear organisation therefore relate to the ontology of the various structural domains that are known – namely how are they related and to what extent are they all aspects of the same entity?

Until recently there has been a lack of comparable, genome-wide chromatin structure data across species and comparative studies have therefore generally examined a single feature of chromatin structure in isolation. Ku et al [20] studied genome-wide Polycomb binding sites and histone modification data in mouse and human embryonic stem (ES cells) within orthologous promoter regions. They stressed the widespread conservation of chromatin states between species, with more than half of promoters showing the same state. Similarly, regions across the orthologous mammalian genome that are enriched for common histone modifications appear to be broadly conserved between human and mouse [21]. In contrast, sequence-specific transcription factor binding patterns appear to evolve rapidly in mammals, with binding events in a particular tissue shared only 10–22% of the time between human, mouse and dog genomes [22]. Higher order chromatin structures are generally assumed to show much less divergence, although detailed studies are rare. The numbers and size distributions of LADs in human lung fibroblasts are reported to be similar to those seen in mouse embryonic fibroblasts, as well as several other mouse cell types [10]. However it is not clear how the extent of divergence between cell types compares with divergence between species, or which genomic regions are involved in either. Replication timing appears generally conserved between human and mouse within large genomic regions showing conserved synteny, but notably less so than between orthologous human and mouse promoters [14]. This conservation has been maintained in spite of the numerous large-scale genome rearrangements separating the two species [23]. It also appears that the similarity in replication timing between species is heavily dependent on the particular cell type examined [14]. On the other hand, Hi-C data has suggested that the mouse and human genomes are separated into largely conserved, megabase sized interaction domains, that are similar between cell types [24].

The studies mentioned above provide complementary views of higher order chromatin structure. Each shows that the mammalian genome is organised into large, discrete domains of higher order chromatin with opposing properties (levels of expression and accessibility, spatial positioning, and replication timing). These domains appear to be broadly similar across the different cells that have been examined, although many regions across the genome show cell type specific structure [8], [10], [14]. However, the actual extent to which these datasets intersect, and how they relate to one another across cell types and species, is poorly understood. Similarly, the genomic loci underlying divergence in chromatin structure between species, and the mechanisms underlying divergence, are unknown. Here we collate a large number of diverse mouse and human datasets to provide the most comprehensive overview of higher order chromatin structure in mammals to date. We undertake a systematic study of all orthologous regions in the mammalian genome and document the extent of conservation in higher order chromatin structure between cell types and during evolution. Our analysis identifies large tracts of structurally divergent chromatin, unevenly distributed across the genome, and containing intriguing enrichments of particular classes of genes.

## Results/Discussion

We conducted our analyses on 36 genome-wide datasets that measure three aspects of higher order chromatin structure and function in mouse and human: replication timing (RT) [7], [14], nuclear lamina association (LA) [9], [10] and genome-wide inter-locus contact preferences (Hi-C) [11], [13]. The datasets were all generated using embryonic or pluripotent cells, with the exception of the Hi-C data (see Methods). All probe-based data were mapped to the latest genome assemblies using UCSC whole genome alignment data (hg19 and mm9), averaged into consecutive non-overlapping 100 Kb regions and collated by their genomic coordinates separately for human and mouse. Orthologous 100 Kb regions were identified conservatively by requiring reciprocal best match overlaps, both at the probe level and 100 Kb region level, between human and mouse genomes (see Methods). This resulted in 16,820 100 Kb regions represented in all higher order structure datasets in both mouse and human genomes. These orthologous regions encompass 54% of the human genome and 62% of the mouse genome. The distributions of the higher order data were examined to ensure global normalisation and scaling was appropriate and quantile normalisation was imposed across all datasets (see Methods). Prior to normalisation all primary datasets showed bimodal distributions with two peaks representing two distinct populations of higher order structure across the mammalian genome (Figure S1), consistent with previous observations [3], [8], [10], [11]. We then addressed two related questions. Firstly, how well do these diverse datasets agree quantitatively? And secondly, what fraction of the mammalian genome can confidently be identified as structurally divergent?

### Widespread conservation of mammalian higher order chromatin structure

**Figure 1:**
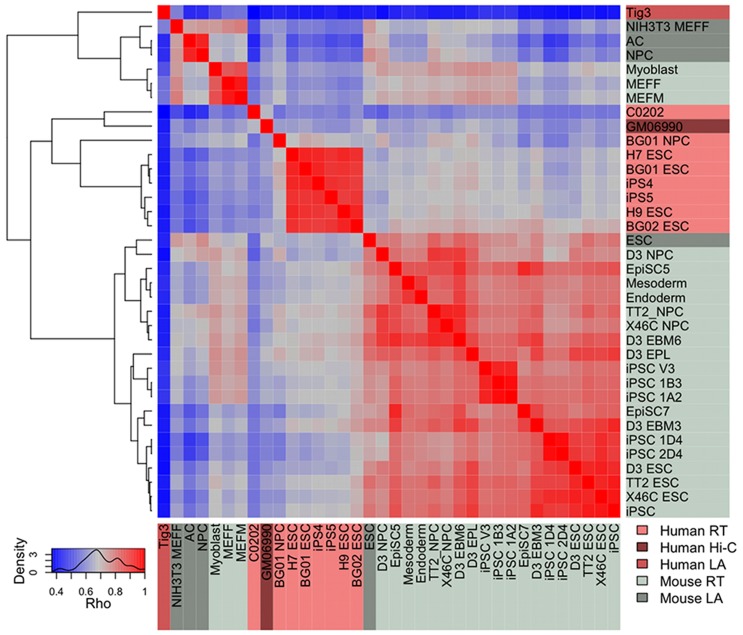
Global correlation matrix of higher order chromatin datasets. The heatmap and dendrogram show the relationships among 36 chromatin structure datasets (Spearman's rho: 0.38 to 0.98, p<1e-16). Datasets are labelled according to the experimental platform of origin: light grey = mouse RT, light pink = human RT, dark grey = mouse LA, medium pink = human LA, dark pink = human Hi-C.

Significant correlations were expected between replication timing (RT), lamin association (LA) and interlocus contact patterns (Hi-C) as they appear to reflect somewhat overlapping aspects of higher order chromatin structure [10], [14], [23]. The degree of agreement overall among the 36 datasets is indeed strong and significant (Spearman's Rho: 0.38 to 0.98, p<1e-16). In spite of differing experimental procedures, platforms, cell types, and species, moderate to strong positive correlations are ubiquitously observed (Figure 1). The highest agreement is usually observed between similar cell types from the same species, even across experimental platforms. For instance mouse RT data for a variety of ES and induced pluripotent stem cell (iPSC) types show strong correlations (Rho: 0.7–0.9, p<2.2e-16) with LAD data from mouse ES cells, and together they form a coherent cluster in the correlation matrix (Figure 1). However, there are also interesting exceptions to this rule, such as the human embryonic fibroblast LA data. Although this dataset shows the weakest correlations to all other datasets, the best agreement is to the mouse fibroblast LA and RT data and not to other human cell types. The reason for this may lie in cell cycle variation: ES and iPS data may be strongly influenced by the fact that these cells are almost entirely in S phase, whereas fibroblasts divide slowly and are mainly in G0/G1. In any case it seems that certain aspects of higher order structure in particular cell types, such as association with the nuclear periphery in fibroblasts, have been more strongly conserved than others during evolution.

**Figure 2:**
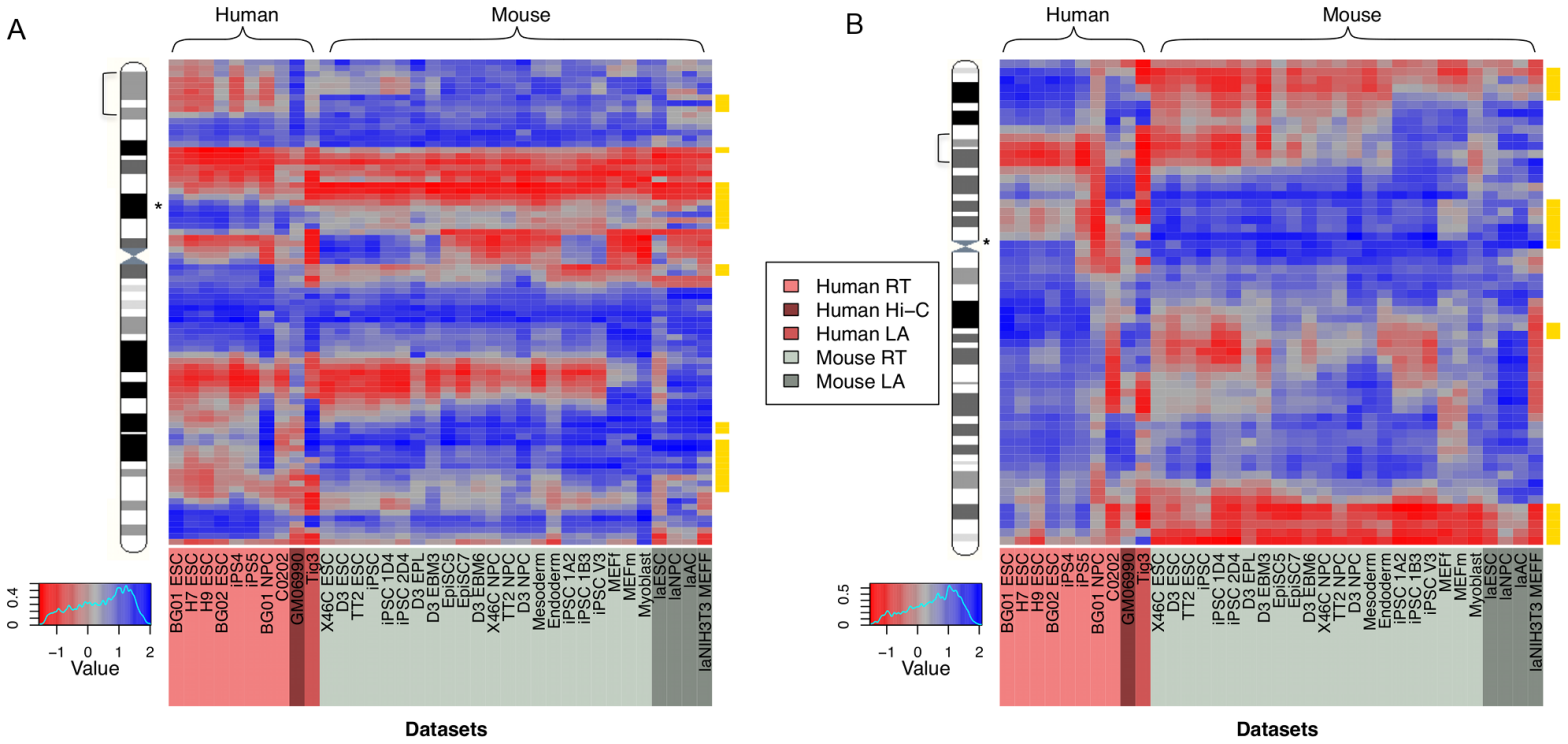
Specific human and mouse regions show significant divergence in higher-order chromatin structure. Human (pink) and mouse (grey) higher order chromatin structure across all cell types assayed, shown for two regions of the human genome: chromosome 11p15.2–15.4 (1.2–15 Mb) with the location of an OR gene cluster indicated by an asterisk (A); chromosome 7p14.3–15.3 (24–32 Mb) with the location of the HOXA gene cluster indicated by an asterisk (B). In each case the chromosome ideogram indicates the region expanded in the heatmaps with a square bracket. Consecutive, orthologous 100 kb regions are positioned on the y-axis with heatmap colours representing relatively open (blue) and closed (red) chromatin structures. Regions displaying significantly divergent chromatin structure are highlighted in yellow.

Striking evidence of structural conservation across the mammalian genome is evident when examining contiguous stretches of orthologous regions (Figure 2). This suggests that many aspects of higher order chromatin structure have been conserved in embryonic cell types, over the ∼80 million years since the divergence of rodents and primates. However apparent divergence in higher order chromatin structure between species is also evident in specific regions. This is most simply seen as loci demonstrating a strong, consistent difference in mean normalised structure between the two species across all of the available datasets (see representative regions depicted in Figure 2). Although there are high correlations between many of these datasets, reflecting similar overall trends in structure as we traverse chromosomes, this can mask substantial variation between datasets at the level of the absolute normalised structural values for a given 100 Kb region (Figure 2). The critical question is therefore, which 100 Kb regions vary between species to an unexpected degree, given the extent of variation seen among all datasets? This is the question we address below using a novel divergence metric based upon permutations of the original data.

We systematically sought genomic regions showing strong and consistent structural divergence between species, across all cell types, using non-parametric tests for each orthologous 100 Kb region (see Methods). The resulting p values were conservatively thresholded to ensure a low false discovery rate (FDR) and robust results. We defined two broad categories of regions based upon their levels of divergence: divergent regions (generating significant p-values passing the FDR threshold) and relatively static non-divergent regions (nonsignificant) (Figure 2; Figure S2). Viewed in this way divergence is necessarily bipolar, containing regions with mean structure values that are relatively open in human but closed in mouse, and vice versa. Such estimates of structural divergence are likely to be inherently conservative, since they depend upon strong consistent evidence for divergence over multiple cell types and experimental platforms. The divergent regions were found to constitute 10.22% (1,719 out of 16,820) of the orthologous regions examined, and possessed a similar (Mann-Whitney test in human p = 0.17, in mouse p = 0.52) protein-coding gene density to non-divergent regions. Human gene densities in nondivergent regions (2.34 per 100 Kb on average) were not significantly different from either human open divergent regions (2.09 per 100 Kb; Mann-Whitney p = 0.45), or human closed divergent regions (2.43 per 100 Kb; Mann-Whitney p = 0.72). Similarly, mouse gene densities in nondivergent regions (1.77 per 100 Kb) were not significantly different from either mouse open divergent regions (1.91 per 100 Kb; Mann-Whitney p = 0.97), or mouse closed divergent regions (1.33 per 100 Kb; Mann-Whitney p = 0.51). The distribution of divergent regions was far from uniform over the genome, with several chromosomes showing higher than expected densities (see Methods; Chi-squared test in human p = 4.34e-06, in mouse p = 1.19e-03). For instance, human chromosomes 5 and 10 were found to have a 50% excess of divergent regions, while chromosomes 21 and 22 were found to have a greater than 60% depletion. This raises the question: does the distribution of divergent regions within chromosomes reflect larger tracts of divergent chromatin?

### Divergent chromatin is clustered within chromosomes

**Figure 3:**
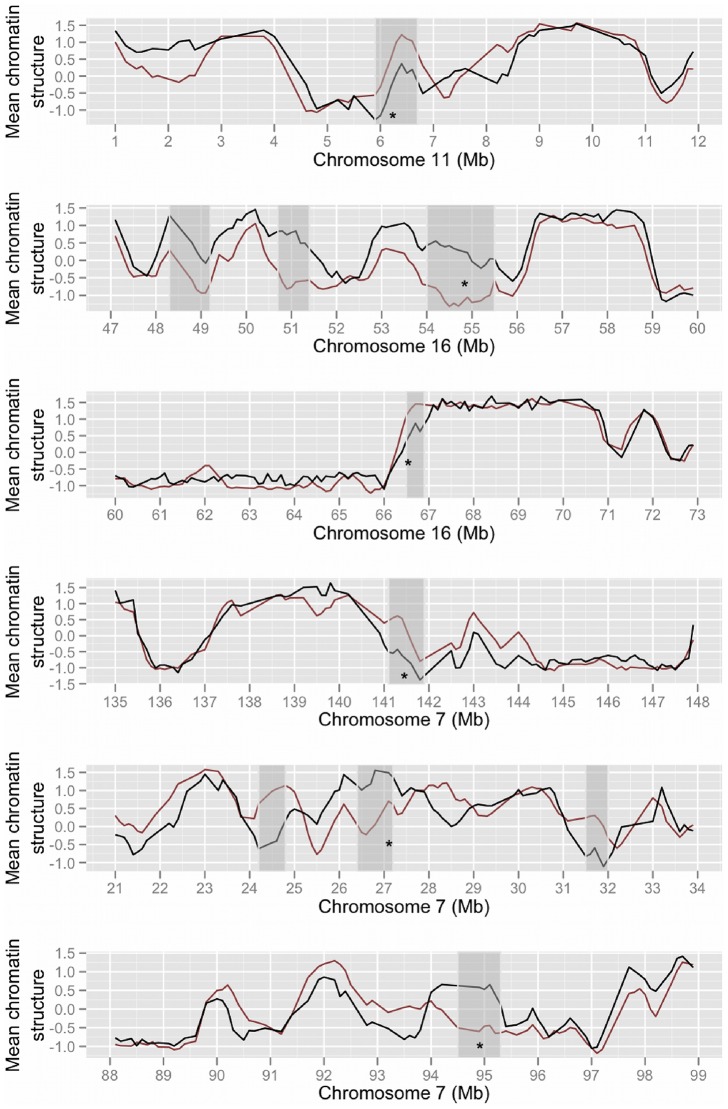
Clustering of divergent chromatin in the human genome. The line plot shows mean normalised human (black) and mouse (red) higher order chromatin structure values across human chromosomes. Unexpectedly large divergent areas are highlighted in grey. Asterisks indicate the positions of functionally enriched gene clusters listed in Table 2.

Cursory examination of these data (e.g. the regions depicted in Figure 2), suggests that a number of divergent 100 Kb regions are clustered in the genome at particular loci. We formally investigated the degree of clustering by measuring the length distribution of consecutive runs of divergent 100 Kb regions observed, relative to the distribution expected using a permutation strategy (see Methods). The clustering observed was found to be highly significant, and we identified 159 unexpectedly large (at least 400 Kb; p<1e-04) clusters of divergent regions with a median size of 800 Kb (Figure 3; Table S2). The same large orthologous clusters were detected in human and mouse genomes when the 100 Kb divergent regions in each genome were clustered (Figure S3), but were not evenly distributed across all chromosomes, for example human chromosomes 3 and 5 had around twice the density expected, but in contrast chromosomes 1 and 9 had around half the density expected. The size distribution of divergence clusters appeared similar to the ES cell chromatin-mediated regulatory domains (median size 880 Kb) recently reported in the mouse and human genomes [24], suggesting that these stretches of divergent chromatin may represent divergent regulatory domains. We therefore examined the similarity in domain boundaries between these regulatory domains and the divergence clusters using a permutation approach (see Methods). An important caveat is the resolution of these datasets, which means that all reported domain boundaries are estimates within tens or hundreds of kilobases. In the human genome the median distance between the boundaries of divergence clusters and the nearest ES cell regulatory domain boundaries was 207,852 bp, which is somewhat less, though not significantly different (p = 0.054) from the expected median distance given 10,000 permuted datasets (235,581 bp). Similarly, in the mouse genome, the equivalent median distance was 260,000 bp, which is not significantly different (p = 0.087) from the expected distance given 10,000 permuted datasets (290,095 bp). We conclude that overall there is no strong association between divergent regions and these regulatory domains, which is consistent with most structural divergence being selectively neutral. We also examined the correspondence between the divergent clusters and regions known to be structurally variable during cellular differentiation from ES cells [7]. Of the 1719 divergent regions, 60 overlapped these structurally dynamic regions, compared with an expected number (mean overlaps in 10,000 permutated datasets) of 99.73 which represents a significant depletion (p<0.013).

The three largest (2.1–2.7 Mb) regions of divergent chromatin were found to occupy subtelomeric regions of human chromosomes 2, 6 and 9 (Figure S4), but in each case the orthologous mouse regions were long distances (80–100 Mb) from mouse telomeres. This was found to reflect the distribution of chromatin divergence across the human genome in general, with unexpected excesses of divergence towards the ends of some human chromosomes (Figure S5; Table S3). This excess was most pronounced within the subtelomeric regions (within 5 Mb of the ends of each chromosome sequence assembly) of 4 human chromosomes (1, 2, 13, 18), and was also seen overall for the human genome (p = 0.016). In contrast most mouse (5 Mb) subtelomeric regions showed a relative depletion of divergence, with none showing significant enrichment, and (nonsignificant) depletion over the mouse genome in general. (No significant enrichment or depletion was found overall for pericentromeric regions in either species.) There are well-characterised differences in the chromatin structures found at human and mouse telomeres, and mammalian telomere biology appears to have been a focus for evolutionary adaptation [25]. Subtelomeric regions are known to be amongst the most rapidly evolving DNA sequences in the genome and have been subject to extensive divergence recently in the primate lineage [26]. The current data suggest that the higher order chromatin structures at some primate subtelomeric regions have also been subject to dramatic change.

**Figure 4:**
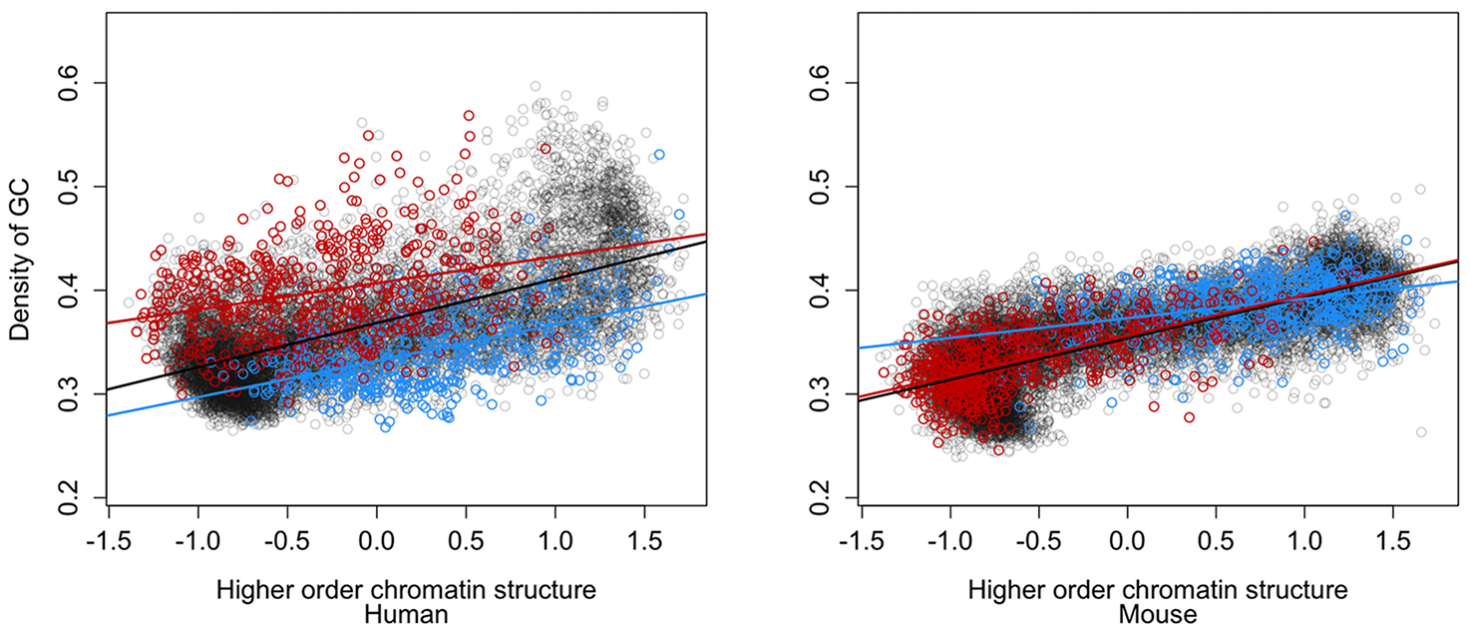
Chromatin divergence and GC content. Percentage of GC nucleotides within all 16,820 100 Kb orthologous regions across the spectrum of mean normalised chromatin structure values. The GC content and higher order structure values for human (left panel) are compared with the GC content and higher order structure values for mouse (right panel). Three classes of regions are shown with their least squares regression lines: nondivergent (grey), divergent open (blue) and divergent closed (red). Note that the bipolar classification of orthologous divergent regions (see text) means that human divergent open regions correspond to mouse divergent closed regions, and vice versa.

Higher order chromatin structure itself is known to show strong positive correlations with GC content, such that relatively open regions are more GC rich and gene dense, and this is also seen here (Figure 4; Human GC density versus chromatin structure Spearman's rho = 0.57, p<2.2e-16; Mouse GC density versus chromatin structure Spearman's rho = 0.75, p<2.2e-16). Similarly, the human genome shows greater variability in GC content overall than in the mouse, consistent with the poor conservation of mammalian isochore structure in rodents [27]. The current data allow us to ask, for the first time, whether GC content is also associated with divergence in higher order structure. Comparison of the percentage of GC nucleotides between divergent and nondivergent regions across all orthologous 100 Kb regions shows intriguing contrasts between the human and mouse genomes (Figure 4). In the human genome there is a significant shift in human GC content between divergent and nondivergent regions, across the entire spectrum of normalised chromatin structure. Furthermore, this shift is to higher GC content (40.5%) within divergent human closed regions, and lower GC content (34.9%) within divergent human open regions, relative to nondivergent regions (37.5%; human divergent open GC versus human nondivergent GC Mann-Whitney p<2.2e-16; human divergent closed GC versus human nondivergent GC Mann-Whitney p<2.2e-16). Thus the two divergence classes show the opposite human GC content bias to the expectation e.g. although open chromatin in the human genome is relatively GC rich (Figure 4), divergent regions that are open in human actually tend to be GC poor. These patterns are not seen in the GC content of the mouse genome, where there is no contradictory shift in the compositional biases of mouse sequences within divergent regions. Instead mouse divergent open regions are relatively GC rich (38.7%) and divergent closed regions are relatively GC poor (33.4%), relative to nondivergent regions (35.5%). Correspondingly there is no global shift in mouse GC content between divergent and nondivergent regions (Figure 4). Thus overall, divergent regions are consistent with the GC content trends seen in the mouse genome, but show a complete contrast with the GC trends in the human genome. The magnitude of the human GC content shift varies between chromatin categories, as reflected in the varying separation between divergent and nondivergent regression lines (Figure 4). Further examination of these data suggests that the largest shifts are seen for regions towards the extreme ends (i.e. unusually open or closed) of the spectrum of chromatin structure categories (Table S1). It is not possible to disentangle cause and effect using the current data, to establish that changes in GC content drive structural change or vice versa. It is also not possible to establish which species has the derived or ancestral chromatin state. However, these observations do suggest that chromatin divergence is often associated with unusual shifts in GC content in the human lineage, which may reflect fluctuations in mutation or selection during primate evolution.

### Chromatin divergence is associated with gene expression divergence in embryonic cells

If genes within divergent regions have undergone regulatory divergence we might expect to see some evidence of this in appropriate expression data. Although perfectly matched expression data is not available, the present data are mainly derived from embryonic cell types and previous studies have examined genome-wide regulatory divergence in human and mouse ES cells. Cai et al (2010) [28] sought significant differences in time-course expression patterns between mouse and human ES cells to rigorously measure regulatory divergence across orthologous genes. They were able to compile classes of genes showing either conserved regulation or divergent regulation in either mouse or human. We examined the distribution of these gene classes across all regions of divergent and nondivergent chromatin. Although the numbers of genes identified by Cai et al (2010) [28] that were also present within the orthologous regions studied here were modest (497 divergent and 126 conserved), we found enrichment (odds ratio: 1.30; Fisher's Exact test p = 0.04) of divergently regulated genes within the 100 Kb regions of divergent higher order chromatin reported here. Genes with conserved regulation were also under-represented in divergent regions (odds ratio = 0.76; p = 0.331). These patterns were observed in spite of the fact that the data of Cai et al (2010) [28] is based upon human and mouse embryonic cell lines that are not represented in the chromatin data studied here. Another more recent study of expression divergence between human and mouse genes, examined expression over a time course in specialised immune (macrophage) cells induced by exposure to bacterial lipopolysaccharide, and reported significant results for larger numbers (186 divergent, 972 conserved) of orthologous gene pairs [29]. We examined these data in the same way and found no significant enrichment of divergently regulated genes in divergent 100 Kb regions. Indeed the genes divergently regulated in these macrophage data showed the opposite trend, and were somewhat under-represented in regions of divergent chromatin (odds ratio: 0.78; p = 0.46). This suggests that the correspondence between chromatin divergence and expression divergence is specific to embryonic cell types.

**Figure 5:**
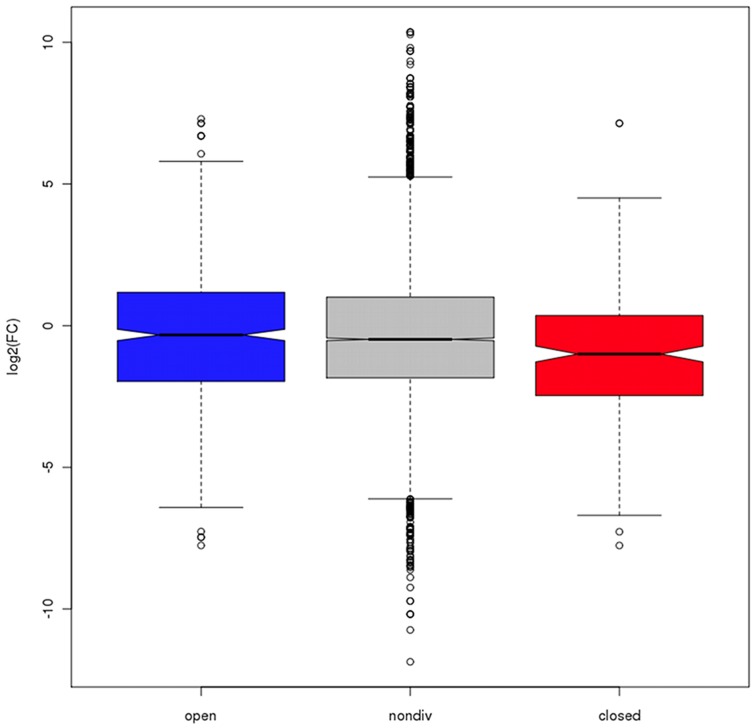
Chromatin divergence and expression divergence. Distributions of log2 fold change (log2(human/mouse expression)) for orthologous gene pairs within nondivergent regions (grey) and two classes of divergent regions: open in human but closed in mouse (blue), closed in human but open in mouse (red). For each class the bottom and top of the box show the lower and upper quartiles respectively around the median, and the width of the notches is proportional to the interquartile range.

We also constructed a larger dataset measuring differential expression between mouse and human ES cells for orthologous gene pairs (see Methods), based upon previous RNAseq studies [30], [31]. These data provide a higher coverage dataset consisting of log fold change measurements for 7,673 gene pairs occurring within the orthologous 100 Kb regions studied here. This allowed us to examine the extent of expression divergence within the two possible bipolar categories of divergent regions, relative to nondivergent regions (Figure 5). We found a striking contrast, with regions open in human but closed in mouse showing a expression divergence consistent with upregulation of human genes (nondivergent median log2 fold change: −0.48; divergent: −0.33; Wilcoxon p = 0.23), while the opposite category (closed in human, open in mouse) showed evidence of upregulation of mouse genes (nondivergent: −0.48; divergent: −1.00; Wilcoxon p = 3.41e-06). This is the pattern of gene expression divergence expected within divergent regulatory domains conferring a respectively permissive or repressive environment for transcription of human genes. Again, these expression data were generated in embryonic cells similar to, but not identical to those used to derive the chromatin divergence data. It is important to note that there is a distinction between the relative bipolar classification of divergent regions (human open/mouse closed and vice versa) and their absolute normalised chromatin values. Thus, it is possible for a region that is relatively open in human and relatively closed in mouse to possess absolute values consistent with a closed conformation in both species. One might expect that using such absolute values to construct more specific divergent region categories might increase the differences seen (Figure 5). This was indeed the case in spite of the associated reductions in sample sizes. Regions open in human but closed in mouse (where the absolute human value > 0 and the absolute mouse value < 0) showed a stronger expression divergence consistent with upregulation of human genes (nondivergent median log2 fold change: −0.48; divergent: 5.03; Wilcoxon p<2.2e-16), while the opposite category (restricted to those with absolute human value<0 and absolute mouse value>0) showed stronger evidence of upregulation of mouse genes (nondivergent: −0.48; divergent: −4.77; Wilcoxon p>2.2e-16). These comparisons to expression data provide independent validation of our methodology and suggest a direct link between the regions of divergent chromatin identified and the regulation of resident genes.

### Regions of divergent chromatin structure harbour developmental gene clusters

Using standard enrichment analyses, we identified over-representation of particular functional classes of genes in the divergent orthologous regions, and the results establish interesting themes. The 907 divergent 100 Kb regions relatively open in human (but closed in mouse) contain 1142 human genes and 757 mouse genes, and both show significant enrichments for multiple terms associated with olfactory receptors (ORs) at particular loci (seen as enrichments for genes mapping to particular cytogenetic bands) (Table 1; Table S2). The mouse genes involved are disproportionately those located in particular OR gene clusters on chromosome 7E3 and 6B1-B2.1, while the human genes are clustered at the orthologous locations at 11p15.4 (Figure 2A) and 7q35 respectively, within extended regions of conserved synteny. Mouse OR genes have been shown to exhibit tightly regulated expression patterns during development, dependent upon repressive chromatin structures spanning clusters of OR genes [32], including histone modifications associated with constitutive heterochromatin [33]. This raises the intriguing possibility of an association between divergent higher order chromatin structures and particular histone modifications. It also suggests that the repressive, relatively closed higher order chromatin structures consistently seen at this region of the mouse genome, but not evident in human cells, could have evolved as part of the regulatory landscape associated with OR gene cluster evolution in rodents.

**Table 1:** The top 5 enriched human and mouse annotation terms for genes within divergent regions of higher order chromatin.

Annotation	Divergent regions	Term	Description	Gene #	P	FDR
Human	Human open/Mouse closed	CYTOBAND	11p15.4	15	1.70E-10	2.17E-07
		GO:0007606	Sensory perception of chemical stimulus	21	2.50E-09	4.15E-06
		GO:0050877	Neurological system process	41	1.42E-07	2.36E-04
		CYTOBAND	10p13	8	3.47E-07	4.44E-04
		GO:0007186	G-protein coupled receptor protein signaling pathway	36	3.81E-07	6.34E-04
Human	Human closed/Mouse open	IPR001827	Homeobox protein, antennapedia type	10	4.80E-07	7.33E-04
		CYTOBAND	18q23	6	5.63E-06	7.52E-03
		GO:0003002	Regionalization	21	8.65E-06	1.50E-02
		CYTOBAND	6q27	6	3.11E-05	4.15E-02
		CYTOBAND	2q37.3	9	3.28E-05	4.38E-02
Mouse	Human open/Mouse closed	GO:0007606	Sensory perception of chemical stimulus	39	2.19E-18	3.58E-15
		GO:0007608	Sensory perception of smell	34	5.80E-16	9.10E-13
		IPR000725	Olfactory receptor	33	7.94E-16	1.15E-12
		GO:0004984	Olfactory receptor activity	33	2.41E-15	3.45E-12
		IPR017452	GPCR, rhodopsin-like superfamily	47	3.73E-15	5.58E-12
Mouse	Human closed/Mouse open	GO:0003002	Regionalization	32	1.96E-09	3.39E-06
		GO:0009952	Anterior/posterior pattern formation	27	2.29E-09	3.97E-06
		GO:0007389	Pattern specification process	36	5.25E-09	9.09E-06
		CYTOBAND	2 45.0 cM	9	1.29E-08	1.89E-05
		CYTOBAND	19 D2	12	3.31E-08	4.84E-05

Other enriched terms include those related to a protocadherin (Pcdh) gene cluster present at 5q31.3 in the human genome, and to the orthologous mouse Pcdh cluster on mouse chromosome 18qB3 (Table S2). Recent work has shown this region adopts distinct chromatin architectures in different mouse neuronal cell types to affect Pcdh gene expression and thereby plays critical roles in establishing neuronal diversity and connectivity during development [34]. A third cluster of genes coincides with this class of divergent regions (open in human, closed in mouse) on mouse chromosome 8D3 (and human 16q21) and is enriched for genes encoding MARVEL, a transmembrane domain involved in membrane apposition. The family of chemokine-like proteins containing this domain have been implicated in inflammation, immunity and development but most are not well characterised. Of the five MARVEL containing genes within the 8D3 divergent cluster, three are unstudied, but Cmtm2a and Cmtm3 are both implicated in the proliferation and development of particular testicular cells [35], [36]. The human ortholog of Cmtm3 is present in the orthologous human divergent region at 16q21 and is a known tumour suppressor gene that shows frequent inactivation via chromatin-mediated silencing in several cancers [37].It seems that developmental gene clusters showing cell type specific regulation are unexpectedly common at regions displaying divergent higher order chromatin. Other clusters of genes, enriched at other divergent regions are also present in the results but lack sufficient functional annotation to generate significant enrichment results after multiple testing corrections (Table S2).

The genes within the divergent 812 orthologous human closed (mouse open) regions contain 1285 human genes and 1102 mouse genes. These also showed significant enrichment for genomic regions harbouring particular gene clusters. Both human and mouse genes in these regions show significant enrichment for terms associated with developmental genes containing Antennapedia type homeobox domains (IPR001827). The genes involved are exemplar developmental genes present at the HOXA (human HOXA1-A7; Figure 2B) and HOXD (human HOXD1-4) clusters. Both clusters are implicated in multiple cancers and other disorders, and are tightly regulated via higher order chromatin domains [38], [39]. It is thought that structural divergence within the chromatin domains harbouring these clusters underlies many important innovations in the vertebrate body plan [40]. Other, relatively poorly studied, homeodomain containing genes at other loci are also present within this class of (human closed, mouse open) divergent regions (Table S2). Again, it seems that developmentally regulated genes are over-represented within regions of divergent chromatin. However, it is worth noting that the proportion of divergent regions generating significant functional enrichments (that is, those divergent regions possessing the genes responsible for the functional enrichments seen) is modest overall, constituting 6% of human and 11% of mouse divergent regions in total.

Most RNA genes are poorly functionally annotated which makes analogous enrichment analyses impossible, but we did examine the densities of the main RNA gene classes (rRNA, snoRNA, snRNA, miRNA, lincRNA) in structurally divergent regions. Only the lincRNA class showed significant differences, with higher densities of both human (divergent mean density: 0.31 genes/Mb; nondivergent mean density: 0.20 genes/Mb; Wilcoxon p = 1.48e-08) and mouse (divergent mean density: 0.12; nondivergent mean density: 0.09; Mann-Whitney p = 3.68e-04) lincRNA genes found in divergent (human closed/mouse open) regions. These molecules are thought to regulate ES cell differentiation via the assembly of chromatin complexes and the establishment of activating or repressive domains [23]. The present data suggest they may also have played roles in chromatin divergence.

As expected the large divergence clusters showed similar patterns of functional enrichments as those discussed above (Table 2; Table S2). For example, the divergent regions mentioned already at 11p15.4 (containing an OR gene cluster) and 16q12.2 (containing an IRX gene cluster) were found to extend across 800 Kb and 1.5 Mb respectively. Similarly the divergent region containing the 7p15.2 HOXA genes was found to encompass 800 Kb, and to include neighbouring lincRNA genes such as HOTAIRM1 which is active in HOXA regulation during neurogenesis and differentiation [41]. An additional region at 7q21.3 showing a novel functional enrichment also emerged, which contains the paraoxonase gene cluster (Table 2), these genes are imprinted in the mouse genome and exhibit unusual, allele-specific expression dependent on developmental stage in human cells [42]. Again, it seems that structural divergence is disproportionately associated with particular developmental gene clusters, which follow tightly regulated expression patterns targeting specific cell types, and are often known to occupy unusual chromatin environments. Many of these genes have also been implicated in developmental adaptations during vertebrate evolution and in human disease processes. This may suggest that regions of divergent chromatin structure have evolved different chromatin conformations to facilitate functional divergence at these loci. However it is not possible to exclude non-adaptive hypotheses, for example where divergence in chromatin structure is a neutral consequence of gene family or repeat expansions or other changes in the underlying genomic sequences. Indeed, since the majority of divergent regions show no detectable functional enrichments, selectively neutral divergence appears to be the most likely scenario in most cases.

**Table 2:** The top 5 enriched human annotation terms for genes within large regions of divergent higher order chromatin.

Cluster	Term	Description	Gene #	P	FDR
chr11:5900000–6699999	CYTOBAND	11p15.4	15	3.74E-28	2.05E-25
	PIRSF038651	G Protein-Coupled Olfactory Receptor, Class I	7	2.76E-10	1.96E-07
	GO:0007608	Sensory Perception Of Smell	8	1.43E-08	2.02E-05
	GO:0007606	Sensory Perception Of Chemical Stimulus	8	6.09E-08	8.59E-05
	IPR000725	Olfactory Receptor	7	6.10E-08	5.77E-05
chr16:54000000–55499999	IPR003893	Iroquois-Class Homeodomain Protein	3	4.26E-07	1.34E-04
	IPR001356	Homeobox	3	2.96E-04	9.32E-02
	IPR017970	Homeobox, Conserved Site	3	3.00E-04	9.45E-02
	IPR012287	Homeodomain-Related	3	3.21E-04	1.01E-01
	CYTOBAND	16q11.2-Q13	2	3.88E-04	1.22E-01
chr16:66500000–66899999	IPR008253	Marvel	5	1.10E-09	7.09E-07
	GO:0042330	Taxis	5	8.79E-07	8.71E-04
	GO:0006935	Chemotaxis	5	8.79E-07	8.71E-04
	GO:0005125	Cytokine Activity	5	6.40E-06	5.41E-03
	GO:0007626	Locomotory Behavior	5	1.04E-05	1.04E-02
chr7:141100000–141899999	CYTOBAND	7q31.3-Q32	3	1.69E-06	7.37E-04
	GO:0008527	Taste Receptor Activity	3	9.84E-06	7.98E-03
	IPR007960	Mammalian Taste Receptor	3	1.02E-05	6.12E-03
	GO:0050909	Sensory Perception Of Taste	3	9.69E-05	9.20E-02
	GO:0007186	G-Protein Coupled Receptor Protein Signaling Pathway	4	2.43E-03	2.28E+00
chr7:26400000–27199999	IPR001827	Homeobox Protein, Antennapedia Type, Conserved Site	7	1.54E-16	4.44E-14
	CYTOBAND	7p15-P14	6	1.16E-13	4.41E-11
	GO:0048562	Embryonic Organ Morphogenesis	7	6.27E-12	7.55E-09
	GO:0009952	Anterior/Posterior Pattern Formation	7	8.06E-12	9.70E-09
	GO:0048568	Embryonic Organ Development	7	2.11E-11	2.55E-08
chr7:94500000–95299999	CYTOBAND	7q21.3	4	3.06E-08	1.17E-05
	GO:0004063	Aryldialkylphosphatase Activity	3	4.22E-07	3.35E-04
	IPR002640	Arylesterase	3	5.11E-07	2.94E-04
	GO:0004064	Arylesterase Activity	3	8.44E-07	6.69E-04
	PIRSF016435	Paraoxonase	3	1.29E-06	1.29E-04

### Conclusions

Individual studies of various aspects of higher order chromatin structure have suggested widespread conservation across the mammalian genome, in spite of many interesting structural differences between cell types [10], [14], [23]. The comprehensive analyses presented here are consistent with this, and demonstrate the same signal across diverse datasets from studies that set out to observe nominally different aspects of structural genome organisation in many different embryonic cell types. We conclude that most measurable aspects of chromatin are conserved across the vast majority of the detectably orthologous genome. However, using a conservative approach (requiring consistent evidence of divergence between species over all cell types and all structural datasets assayed) we also observe divergent chromatin structure at 10.22% of orthologous 100 Kb genomic regions examined, encompassing over 170 Mb and including many hundreds of human and mouse genes. This suggests that structural divergence has played a major role in the evolution of many loci occupying these unusual genomic regions. Many of the regions identified form unexpectedly large tracts of divergent chromatin, nonrandomly distributed between and within chromosomes, and this clustering appears particularly pronounced at human subtelomeric regions. Overall the divergent regions of embryonic chromatin identified are significantly enriched for genes active in vertebrate development. These include homeodomain gene clusters, which have been implicated in evolutionary innovations to vertebrate developmental programmes, suggesting that selection may have modulated their regulation during evolution via alterations to chromatin. Consistent with this we find that genes showing evidence of regulatory divergence between human and mouse are over-represented within regions of divergent higher order chromatin structure.

The mechanisms underlying divergence in higher order chromatin structure remain unknown, but one may speculate that alterations at lower levels of chromatin are likely to be involved. For example, changes in the diversity or abundance of relatively rapidly evolving ncRNAs, which can mediate chromatin remodelling between cell types [43], could provide a molecular basis for divergence. Also the strong sequence-level correlates of human chromatin structure [44], [45] and the unusual, lineage specific shifts in GC content seen here, suggest it is possible that sequence divergence underlies chromatin divergence. It may also be relevant that larger scale variation in chromatin structure within the mammalian genome is often associated with alterations in the spectrum of histone modifications at a region. For example, human LADs are reported to show enrichments of H3K9 and H3K27 methylation [46], and OR gene clusters are now known to possess an unusual signature of histone modifications involving the molecular hallmarks of constitutive heterochromatin [33]. It is therefore possible that divergence in chromatin domains during evolution is caused by alterations in the constellations of histone modifications present. However, definitive evidence of the mechanisms underlying evolutionary divergence in higher order chromatin structure will require substantial future investigations.

## Methods

### Higher order chromatin structure data

All cell types and datasets, and their abbreviations are listed in Table S5. Replication timing data in human and mouse embryonic cells were obtained from Hiratani et al [7], and Ryba et al [14] as log2(early relicating/late replicating) values. Nuclear lamina association data in human and mouse embryonic cells were obtained from Guelen et al [9] and Peric-Hupkes et al [10]. Both studies were based upon the DamID technique for labelling lamina associated sequence, where relative lamina association is represented by log2(Dam-fusion/Dam-only) values. Finally, 100 Kb window genomic interaction probability matrix eigenvalues were defined for human lymphoblastoid cells using Hi-C by Lieberman-Aiden et al [11]. These values were found to largely reflect two relatively open and closed nuclear compartments of higher order chromatin. Although these data were not derived from embryonic cells it appears that many of the higher order patterns (as represented by interaction matrix eigenvectors) in Hi-C datasets are consistent between cell types [11], [24]. Re-analysis of these interaction data has revealed the presence of systematic biases that afflict the Hi-C method, obscuring additional, finer scale structural compartments [12]. Although our analysis only concerns the course grained, two-compartment division between open and closed regions (since we use eigenvalues of interaction matrices not interaction probabilities themselves) we were concerned that our results might be affected by these biases. Consequently we examined an independent genomic interaction map produced for a similar lymphoblastoid cell line using a modified Hi-C method designed to mitigate the biases inherent in previous data [13]. When the original [11] interaction data were substituted with these new, nominally unbiased [13] data we observed very similar correlations with all other chromatin structure datasets. We conclude that the biases present in the Lieberman-Aiden et al [11] dataset have little effect on a course grained, two compartment classification of the genome based upon these data, and therefore that our search for structurally divergent regions is unaffected.

### Orthology and divergence

Probe based replication timing and nuclear lamina association data coordinates were translated to the latest human or mouse genome assembly coordinates (hg19 and mm9) using reciprocal liftOver transformations to ensure accurate remapping [47]. Probes failing to map reciprocally to overlapping coordinates between mouse and human genomes were discarded as unreliable. For each dataset the structural data values were averaged across probes into consecutive non-overlapping 100 Kb regions, but regions represented by fewer than 10 probes were discarded as potentially unreliable. This allowed comparisons between the probe based datasets and the Hi-C data, which has a fixed resolution of 100 Kb. Within each species 100 Kb regions were collated across datasets where their coordinates overlapped by 50% or more. The result was a set of 24,711 mouse and 28,786 human 100 Kb regions represented by higher order structural values from multiple datasets. Orthologous 100 Kb regions were defined as those regions with at least a 50% coordinate overlap between mouse and human genomes using reciprocal liftOver transformations. A total of 16,820 100 Kb orthologous regions, covering 54% of the human genome and 62% of the mouse genome, were defined in this way. A total of 11,966 human and 7,891 mouse regions, lacking an orthologous mapping using this protocol, were designated putatively lineage specific regions. As expected, lineage specific regions were highly enriched for segmental duplications, repeats and duplicated gene families, whereas orthologous regions were relatively rich in protein coding genes [48]. Examination of several techniques revealed that standard quantile normalisation procedures (R/Bioconductor limma package) [49] used to normalise across different microarray experiments were effective across the different experimental platforms and cell types here, therefore this normalisation technique was implemented across all structural datasets for all 100 Kb regions (Figure S1; Figure S7). The normalised structural data and chromosome coordinates for all 16,820 orthologous regions are provided in Table S6.

Structurally divergent regions were defined as orthologous 100 kb regions that showed a consistent difference in higher order structural values across human and mouse data. Non-parametric tests from the SAM package [50], analogous to two class unpaired t-tests with permutation derived p-values, were used to assess divergence (R package samr). These tests were developed for microarray data analysis but are appropriate for other types of non-microarray derived data [50]. The approach was developed to identify unusual genes that show a strong and consistent expression difference between treatments, given many variable replicate measurements. In the present case we identify unusual 100 Kb regions, showing a strong and consistent difference between species, given the many variable measurements of chromatin structure. In both cases the aim is to identify significant differences between states (treatments, species) for the measured entities (genes, 100 Kb regions) given a number of inherently noisy, variable observations. The permutation approach ensures that the observed variability in the observations is accounted for in the significance of the test result. Tests were carried out for each 100 Kb orthologous region, with the various normalised structural values for that region compared between species. 100,000 permutations of the normalised structure dataset were used to estimate the false discovery rate (FDR), defined in this instance as the median number of false positive divergent regions expected (given the permuted datasets), divided by the total number of divergent regions called. The FDR threshold was set to be relatively low (FDR = 2e-04) to ensure that less than 1 false positive was expected within the 1719 divergent regions found. The results are necessarily bipolar with positive and negative divergent regions called to indicate human open/mouse closed or human closed/mouse open divergence respectively. Relatively static, nondivergent regions were classed as those with p values that did not pass the FDR threshold. The mean normalised structure values for 100 Kb regions, over all of the available datasets in a species, were calculated as a useful guide to trends in structure across chromosomes and the genome overall.

The 100 Kb detectably orthologous regions defined above (using a 50% overlap threshold) will necessarily vary in the degree of similarity they show between species, it was therefore a concern that this might influence the measurement of structural divergence. Specifically it was important to show that the regions identified as structurally divergent are not simply those most poorly aligned between species at the sequence level. On closer examination the distributions of overlaps (aligned nucleotides minus gaps) were found to be very similar between structurally divergent and nondivergent regions, whether viewed in terms of the human (hg19) genome (divergent overlap mean = 0.80, median = 0.81; nondivergent overlap mean = 0.79, median = 0.80), or the mouse (mm9) genome (divergent overlap mean = 0.73, median = 0.72; nondivergent overlap mean = 0.72, median = 0.71) sequence assemblies, based upon UCSC whole genome alignments. We concluded that our estimates of structural divergence are not a simple reflection of sequence divergence.

### Distribution and gene content of divergent regions

We examined the distribution of divergent regions across chromosomes by comparing the expected numbers, given the proportion of orthologous 100 Kb regions on each chromosome, with those observed using chi-squared tests, and identified chromosomes of interest as those generating standardized residuals>1.96. To define divergence clusters (i.e. clustered groups of divergent 100 Kb regions) we first identified all consecutive runs of significantly divergent regions across the orthologous human (and separately the mouse) genome, and the observed distribution of their lengths. Consecutive runs were required to maintain the polarity of divergence (i.e. all regions involved must be either human open/mouse closed or vice versa). We then permuted the divergence data among orthologous 100 Kb regions within chromosomes 10,000 times, and noted the length distributions of consecutive runs within each permuted genome. The frequency with which a run of n consecutive divergent 100 Kb regions was seen in the permuted datasets was taken as an approximate p value for runs of length n in the observed dataset. Observed runs of divergent regions greater than or equal to 400 Kb were never seen in the permutated data (p<0.0001) and were taken to be significant divergence clusters. This strategy is likely to be conservative in detecting large regions of divergent chromatin as it does not allow for gaps (e.g. regions that may have marginally failed to reach significance in the test for divergence above) within runs of divergent regions. 159 large divergent regions were discovered at the same, orthologous locations in the human and mouse genomes (Table S3). An additional 1.4 Mb divergent region (at chr18: 11600000–12999999) was found in the mouse genome that lacked a reciprocally orthologous human region.

Enrichment or depletion of 100 Kb divergent regions within subtelomeric or centromeric regions was assessed using a circular permutation strategy [51] to preserve the observed degree of clustering, over 10,000 permuted datasets. Each permuted dataset was generated by shifting the locations of all divergent regions on each chromosome by a random number (less than the length of the chromosome). Regions assigned a shifted position greater than the final base pair of the chromosome are reassigned to the start of that chromosome (plus the number of bases by which they exceeded the final base pair). Thus the permutations regard chromosomes as circularised, and thereby maintain the degree of clustering seen among the observed divergent regions. The number of permuted datasets, n, possessing a number of divergent regions within subtelomeric (or centromeric) regions greater than or equal to the observed number were noted, and used to calculate approximate p-values (n/10,000) for enrichment. The significance of depletion was calculated analogously, according to the number of permuted datasets possessing the same or fewer divergent regions. Subtelomeric regions were defined as regions within 1 Mb, 5 Mb and 10 Mb of the first and final base pairs of the chromosome assemblies, and within the final base pair of the (acrocentric) mouse assemblies. Centromeric regions were defined as regions within 1 Mb, 5 Mb and 10 Mb of the first base pair of mouse and human chromosome q arm assemblies, and within the final base pair of human p arm assemblies. It is important to note that the density of orthologous 100 Kb regions within subtelomeric regions was not significantly different from the genome as a whole, either for human (5 Mb subtelomeric region mean density = 23.70; mean density across all genomic 5 Mb bins = 28.10) or mouse (5 Mb subtelomeric region mean density = 34.60; mean density across all genomic 5 Mb bins = 34.20). The same circular permutation approach was used to measure the enrichment or depletion of divergent regions within domains that are structurally dynamic during cellular differentiation [7]. We also used a similar permutation strategy to compare the similarity (i.e. proximity) of domain boundaries between chromatin-mediated regulatory domains [24] and the boundaries of divergent clusters. The median distance between divergent cluster boundaries and the nearest regulatory domain boundaries was compared to the median distance seen in 10,000 datasets that had undergone circular permutation. The proportion of datasets generating a median distance less than or equal to the observed median distance was taken as an approximate p-value.

Gene densities were calculated per Mb for divergent and nondivergent datasets and tested using nonparametric (Mann-Whitney/Wilcoxon test) statistics. Functional enrichments for protein coding genes were calculated using DAVID [52] using the total human and mouse genes present within the 16,820 orthologous 100 Kb regions as background sets for human and mouse enrichment analyses respectively. Enrichment of each annotation term in the set of human or mouse genes present within divergent regions was assessed using default options (p-values calculated using the hypergeometric distribution with FDR correction). Enrichment of these gene sets within cytogenetic bands was also examined as this can reflect the clustering of divergent regions. Both protein coding and RNA genes were annotated by Ensembl (http://www.ensembl.org) and include lincRNAs predicted according to combinations of histone modifications and complementary EST and cDNA data. RPKM expression values for human H1 ES cells [30] and mouse E14 ES cells [31] were used to calculate log2(human RPKM/mouse RPKM) for all one to one orthologous mouse human Ensembl gene pairs, as an estimate of fold change in expression.

## Supporting Information

Figure S1 **Structural data distributions.** The bimodal distributions of higher order structural data for all orthologous 100 Kb regions before normalisation with two peaks representing two distinct populations of higher order structure across the mammalian genome. Human and mouse RT data, LA data, and human Hi-C data are shown.
(JPG)

Figure S2 **Quantifying human-mouse divergence in higher-order chromatin structure.** The Q-Q plot from the two class unpaired SAM tests (see Methods) for each orthologous 100 Kb region. Significantly divergent regions (highlighted in green and red) generate unexpectedly extreme observed test scores relative to the expected (permutation based) scores.
(JPG)

Figure S3 **Distribution of mammalian divergence clusters.** Large human divergent regions (red) are shown with the orthologous positions of large mouse (blue) divergent regions in the human genome.
(JPG)

Figure S4 **The three largest divergence clusters on human chromosomes.** The line plot shows mean normalised human (black) and mouse (red) higher order chromatin structure across human chromosomes. Unexpectedly large divergent areas are highlighted in grey.
(JPG)

Figure S5 **Distribution of structural divergence across the human and mouse genomes.** The occurrence of divergent orthologous 100 Kb regions across human (top panel) and mouse (bottom panel) chromosomes. In each species the divergent regions found to be relatively open (blue) or relatively closed (red) within that species are indicated.
(JPG)

Figure S6 **Enriched functional classes within divergent regions.** The relationships between enriched GO terms for genes within divergent 100 Kb regions, related terms are coloured similarly and the areas ascribed to each term reflect the significance of their enrichment.
(JPG)

Figure S7 **Structural data distributions after normalisation.** The identical bimodal distributions of higher order structural data across all orthologous 100 Kb regions, after quantile normalisation. Representative datasets of human (BG01) and mouse (iPSC V3) RT data, human (Tig3) and mouse (NIH3T3) LA data, and human Hi-C data (GM06990) are shown, both separately and together (All).
(JPG)

Table S1 **GC content and structural divergence.** Percentage of GC nucleotides within all 16,820 100 Kb orthologous regions across the spectrum of normalised chromatin structure values as in Figure 4. The GC content difference between divergent and nondivergent regions is shown for each binned category of higher order structure, together with the significance of the difference according to Mann-Whitney tests.
(DOCX)

Table S2 **Full functional enrichment results.** Functional enrichment results for all classes and clusters of divergentregions.
(XLS)

Table S3 **Full divergent region details.** All divergent orthologous regions discovered.
(XLS)

Table S4 **Enrichment of divergence clusters at subtelomeric regions.** Results of permutation tests (see Methods) assessing the significance of observed relative to expected numbers of divergence clusters at a variety of proximities (1 Mb, 5 Mb, 10 Mb) to telomeres in human and mouse genomes. Significant (p<0.05) enrichments (labelled E) or depletions (labelled D) in observed relative to expected numbers are highlighted in yellow.
(XLS)

Table S5 **Cell types and datasets.** Details of the cell lines, data types and embryonic stages in this study.
(DOC)

Table S6 **Full orthologous region details.** Structural data for all orthologous regions examined.
(CSV)
