# Supplementary figures and images for: Divergence of Mammalian Higher Order Chromatin Structure Is Associated with Developmental Loci
Source: PLoS Comput Biol. 2013 Apr 4;9(4):e1003017. doi: 10.1371/journal.pcbi.1003017 (PMC3617018; doi:10.1371/journal.pcbi.1003017)

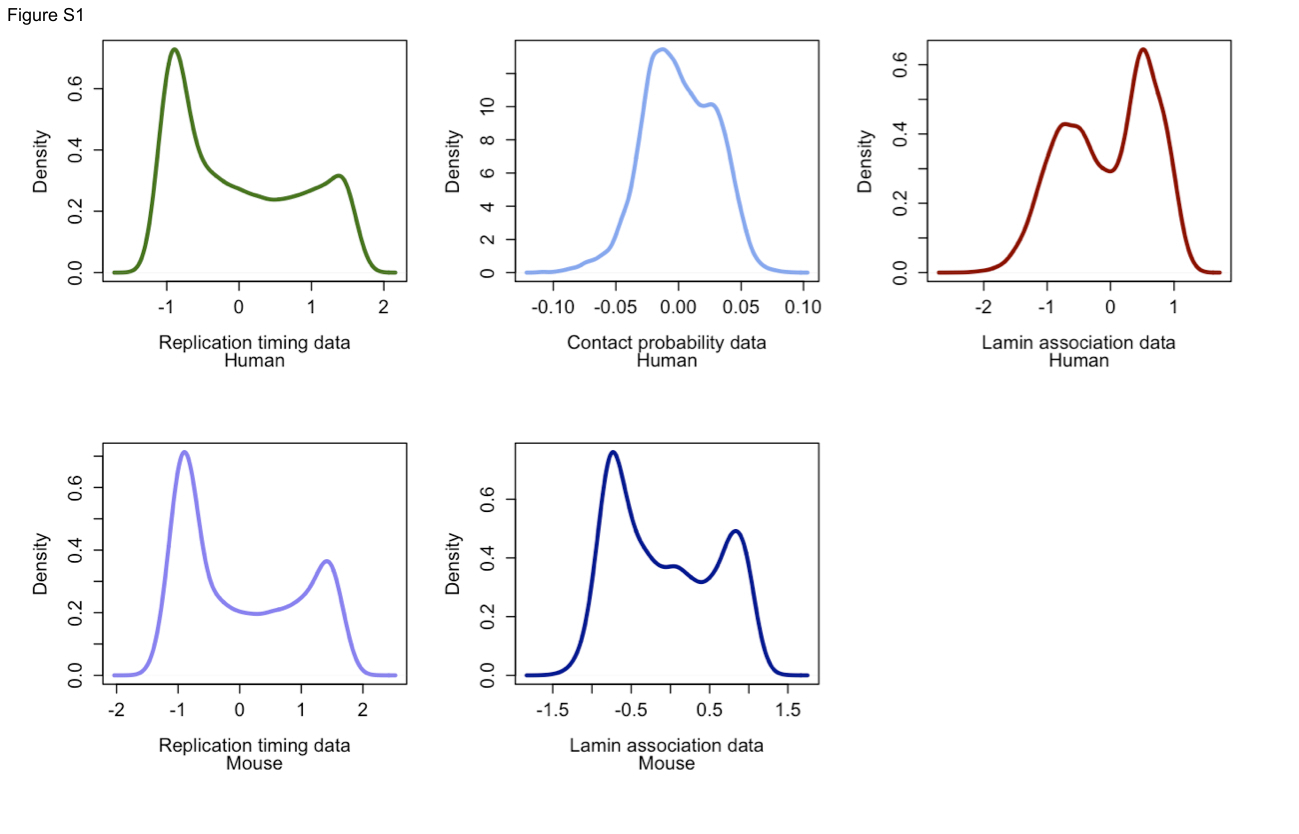

Supplement: Figure S1 — Structural data distributions. The bimodal distributions of higher order structural data for all orthologous 100 Kb regions before normalisation with two peaks representing two distinct populations of higher order structure across the mammalian genome. Human and mouse RT data, LA data, and human Hi-C data are shown. (JPG) [file pcbi.1003017.s001.jpg]

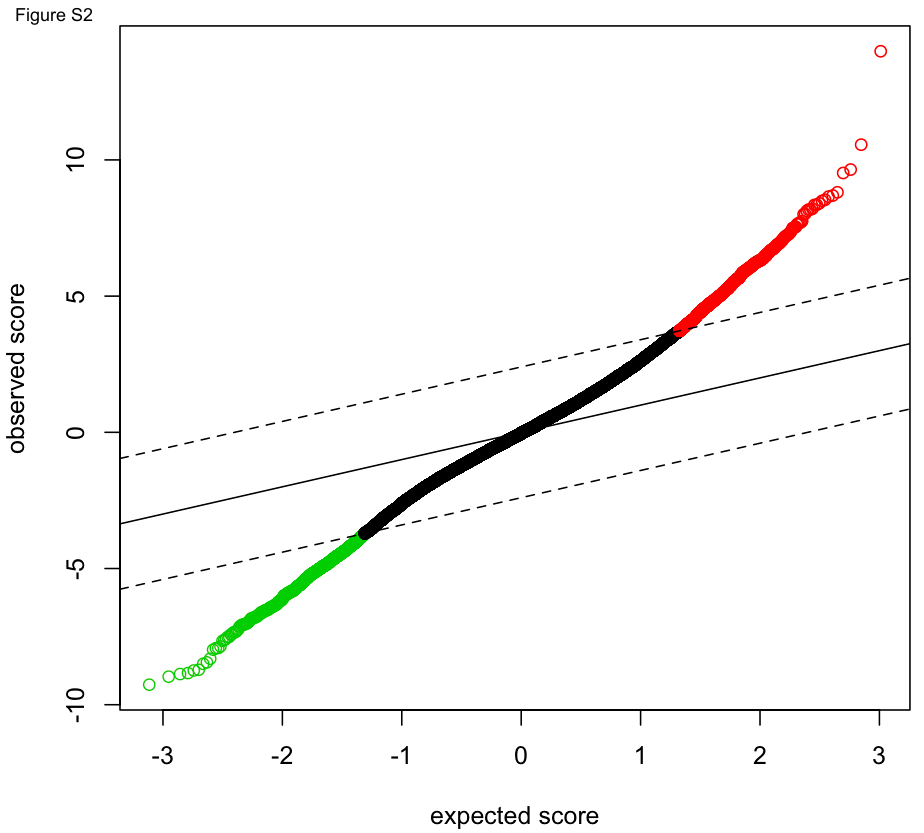

Supplement: Figure S2 — Quantifying human-mouse divergence in higher-order chromatin structure. The Q-Q plot from the two class unpaired SAM tests (see Methods) for each orthologous 100 Kb region. Significantly divergent regions (highlighted in green and red) generate unexpectedly extreme observed test scores relative to the expected (permutation based) scores. (JPG) [file pcbi.1003017.s002.jpg]

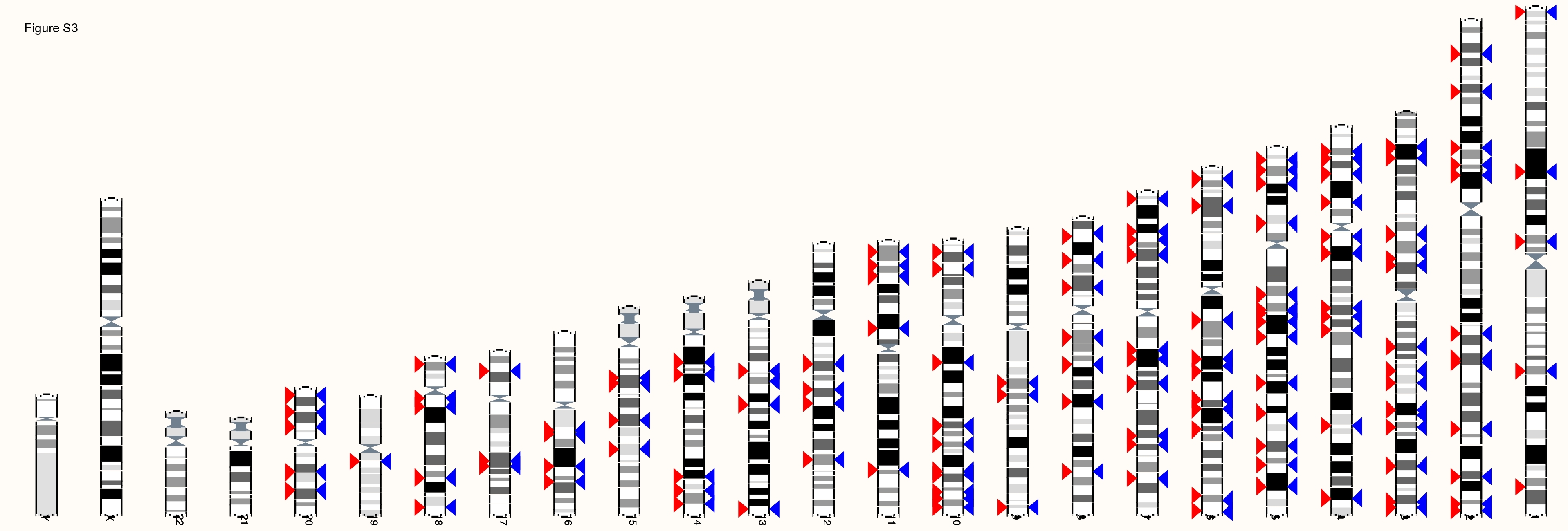

Supplement: Figure S3 — Distribution of mammalian divergence clusters. Large human divergent regions (red) are shown with the orthologous positions of large mouse (blue) divergent regions in the human genome. (JPG) [file pcbi.1003017.s003.jpg]

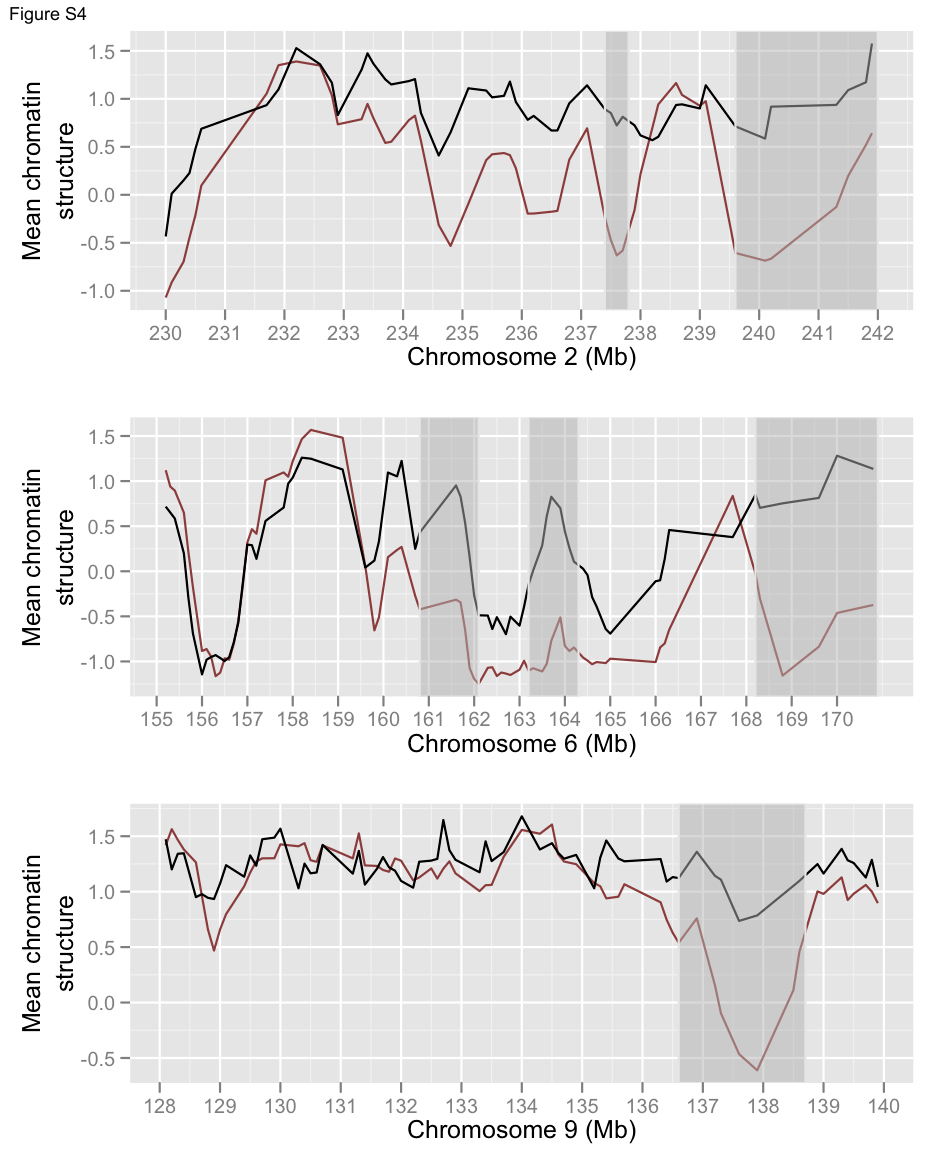

Supplement: Figure S4 — The three largest divergence clusters on human chromosomes. The line plot shows mean normalised human (black) and mouse (red) higher order chromatin structure across human chromosomes. Unexpectedly large divergent areas are highlighted in grey. (JPG) [file pcbi.1003017.s004.jpg]

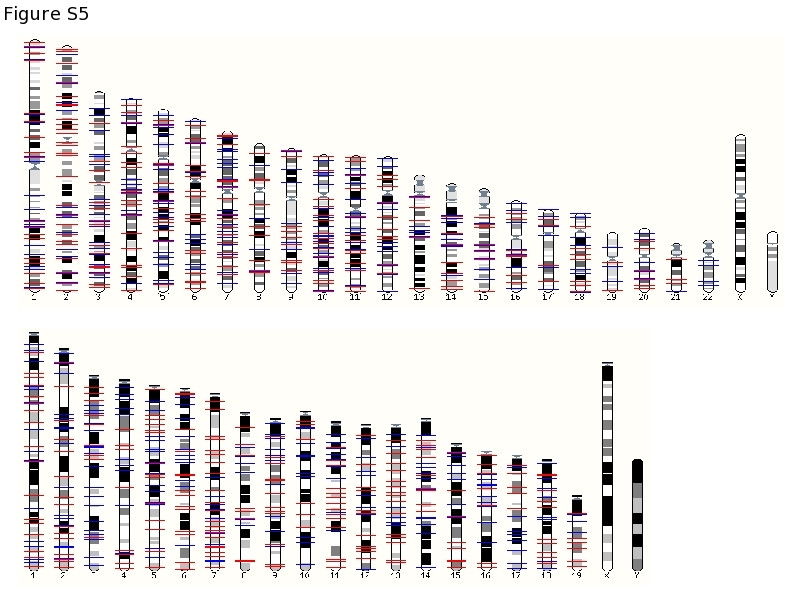

Supplement: Figure S5 — Distribution of structural divergence across the human and mouse genomes. The occurrence of divergent orthologous 100 Kb regions across human (top panel) and mouse (bottom panel) chromosomes. In each species the divergent regions found to be relatively open (blue) or relatively closed (red) within that species are indicated. (JPG) [file pcbi.1003017.s005.jpg]

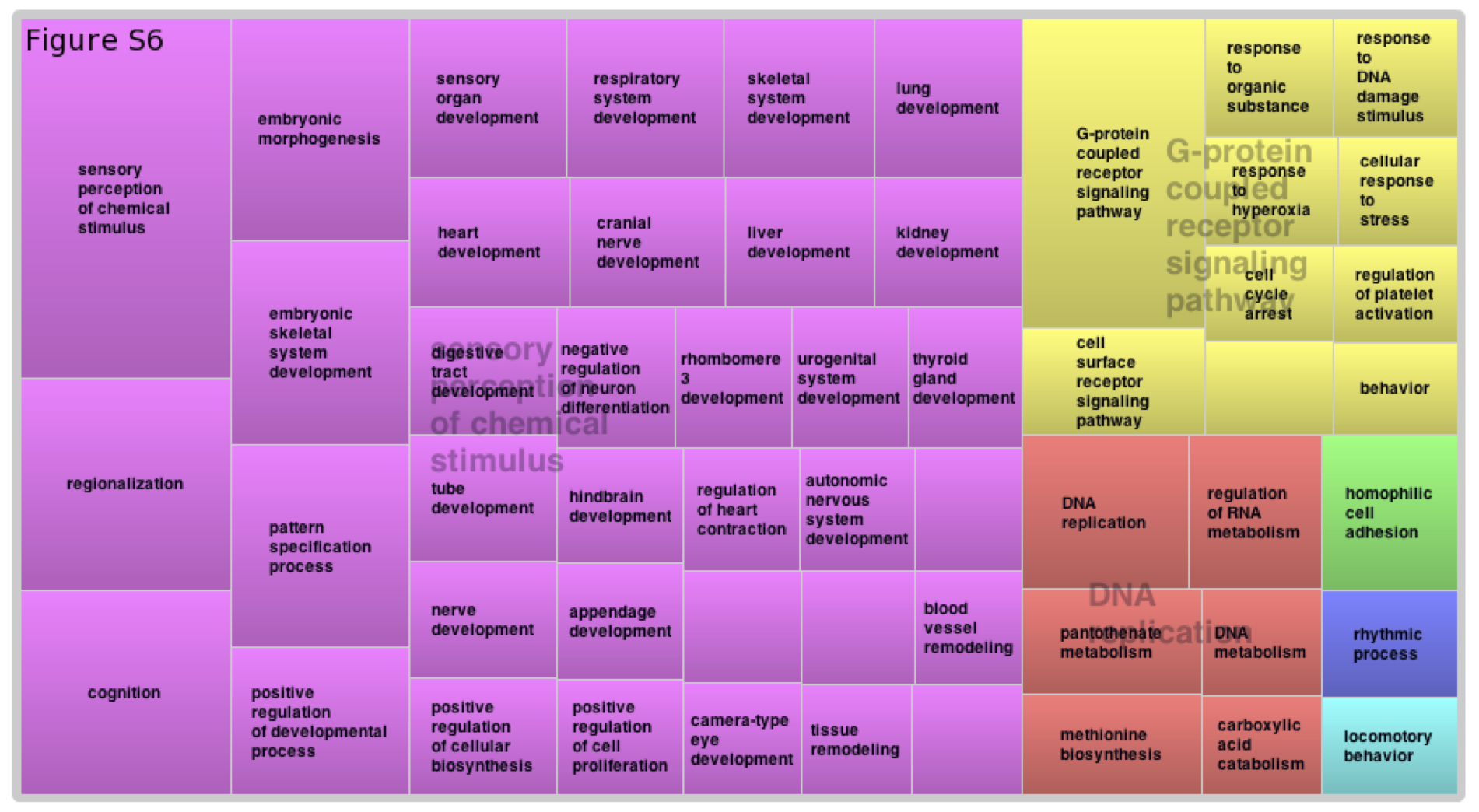

Supplement: Figure S6 — Enriched functional classes within divergent regions. The relationships between enriched GO terms for genes within divergent 100 Kb regions, related terms are coloured similarly and the areas ascribed to each term reflect the significance of their enrichment. (JPG) [file pcbi.1003017.s006.jpg]

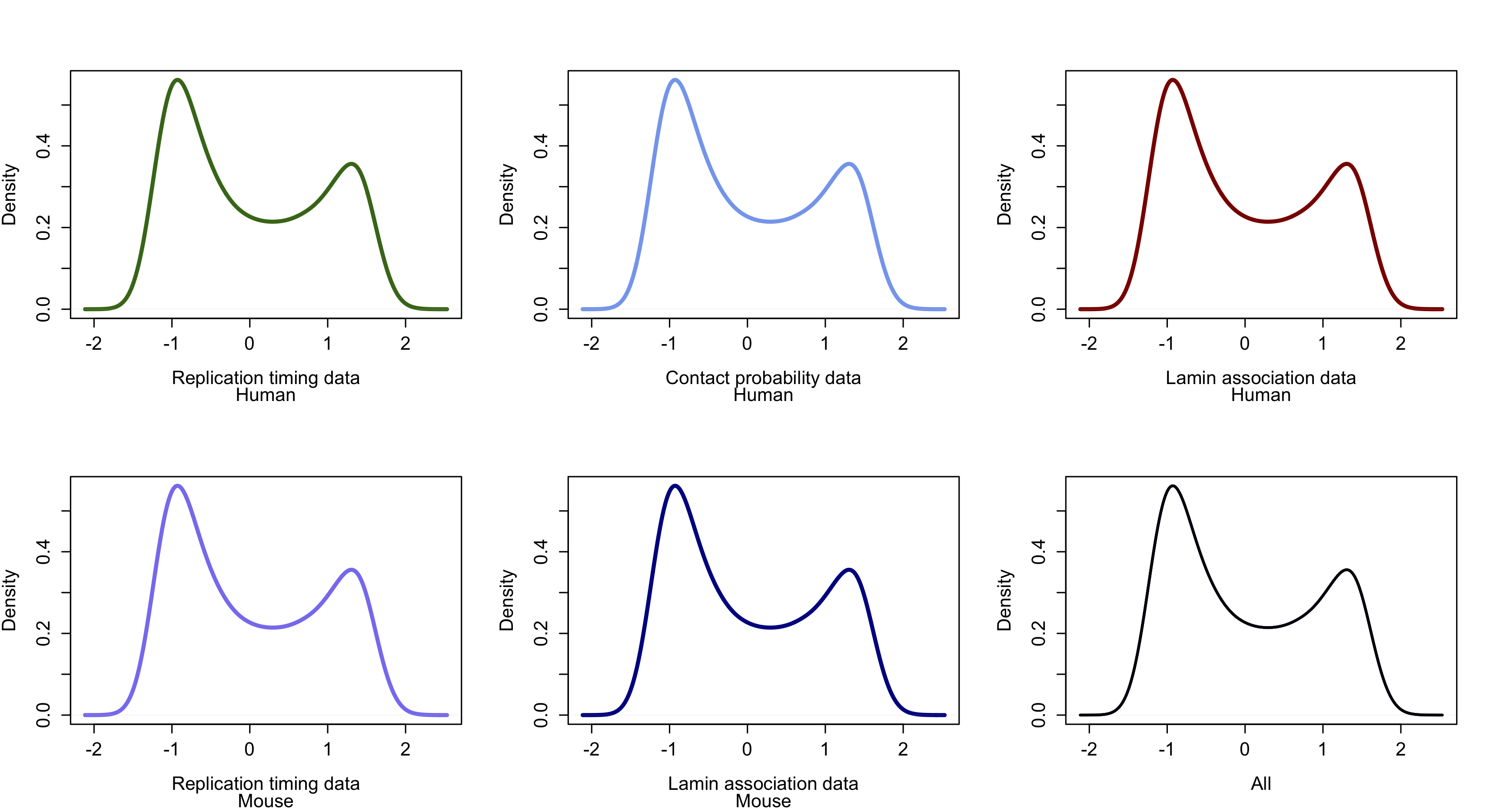

Supplement: Figure S7 — Structural data distributions after normalisation. The identical bimodal distributions of higher order structural data across all orthologous 100 Kb regions, after quantile normalisation. Representative datasets of human (BG01) and mouse (iPSC V3) RT data, human (Tig3) and mouse (NIH3T3) LA data, and human Hi-C data (GM06990) are shown, both separately and together (All). (JPG) [file pcbi.1003017.s007.jpg]
