# Supplementary material for: Divergence of Mammalian Higher Order Chromatin Structure Is Associated with Developmental Loci
Source: PLoS Comput Biol. 2013 Apr 4;9(4):e1003017. doi: 10.1371/journal.pcbi.1003017 (PMC3617018; doi:10.1371/journal.pcbi.1003017)
Supplement: Table S1 — GC content and structural divergence. Percentage of GC nucleotides within all 16,820 100 Kb orthologous regions across the spectrum of normalised chromatin structure values as in Figure 4. The GC content difference between divergent and nondivergent regions is shown for each binned category of higher order structure, together with the significance of the difference according to Mann-Whitney tests. (DOCX) [file pcbi.1003017.s008.docx]

Table S1

| Structure | -1.2 | -1 | -0.8 | -0.6 | -0.4 | -0.2 | 0 | 0.2 | 0.4 | 0.6 | 0.8 | 1 | 1.2 | 1.4 |
| --- | --- | --- | --- | --- | --- | --- | --- | --- | --- | --- | --- | --- | --- | --- |
| Human | | | | | | | | | | | | | | |
| Non-divergent | 0.358 | 0.335 | 0.324 | 0.333 | 0.351 | 0.356 | 0.365 | 0.372 | 0.378 | 0.388 | 0.397 | 0.422 | 0.432 | 0.441 |
| Divergent | 0.381 | 0.381 | 0.378 | 0.374 | 0.363 | 0.365 | 0.364 | 0.365 | 0.364 | 0.387 | 0.372 | 0.373 | 0.368 | 0.388 |
| Delta GC | 0.023 | 0.046 | 0.054 | 0.041 | 0.012 | 0.009 | -0.001 | -0.006 | -0.014 | -0.001 | -0.025 | -0.049 | -0.064 | -0.053 |
| P | 1.20E-03 | 1.33E-15 | 8.39E-19 | 7.08E-13 | 1.54E-01 | 8.59E-01 | 1.93E-01 | 1.10E-01 | 4.38E-03 | 6.48E-01 | 3.57E-03 | 6.29E-07 | 3.64E-08 | 2.09E-05 |
| Mouse | | | | | | | | | | | | | | |
| Non-divergent | 0.328 | 0.319 | 0.315 | 0.326 | 0.351 | 0.360 | 0.364 | 0.369 | 0.373 | 0.378 | 0.380 | 0.392 | 0.405 | 0.403 |
| Divergent | 0.322 | 0.311 | 0.309 | 0.321 | 0.349 | 0.363 | 0.368 | 0.370 | 0.383 | 0.374 | 0.388 | 0.389 | 0.407 | 0.407 |
| Delta GC | -0.007 | -0.008 | -0.006 | -0.005 | -0.001 | 0.003 | 0.004 | 0.001 | 0.010 | -0.004 | 0.009 | -0.003 | 0.002 | 0.003 |
| P | 2.55E-01 | 2.13E-02 | 7.12E-02 | 3.03E-01 | 7.87E-01 | 6.13E-01 | 4.33E-01 | 7.28E-01 | 7.83E-02 | 3.76E-01 | 1.41E-01 | 3.90E-01 | 8.25E-01 | 5.74E-01 |
