# Supplementary material for: Divergence of Mammalian Higher Order Chromatin Structure Is Associated with Developmental Loci
Source: PLoS Comput Biol. 2013 Apr 4;9(4):e1003017. doi: 10.1371/journal.pcbi.1003017 (PMC3617018; doi:10.1371/journal.pcbi.1003017)
Supplement: Table S5 — Cell types and datasets. Details of the cell lines, data types and embryonic stages in this study. (DOC) [file pcbi.1003017.s012.doc]

Table S3

| **Study** | **Data Type** | **Species** | **Developmental Stage** | | **Cell Type** |
| --- | --- | --- | --- | --- | --- |
| Hiratani et al. 2009 | Replication Timing Log2(Early/Late) | Mouse | Early Embryonic | ESC/IPC | X46C-ESC, D3-ESC, TT2-ESC, iPSC |
|  |  |  |  | Early Epiblast | D3-EPL, D3-EMB3 |
|  |  |  |  | Late Epiblast | EpiSC5, EpiSC7 |
|  |  |  |  | Ectoderm | D3-EBM6, X46C-NPC, TT2-NPC, D3-NPC |
|  |  |  |  | Mesoderm | Mesoderm |
|  |  |  |  | Endoderm | Endoderm |
|  |  |  | Late Embryonic | Late Mesoderm | MEFF, MEFM, Myoblast |
| Ryba et al. 2010 | Replication Timing Log2(Early/Late) | Human | Early Embryonic | ESC | BG01, BG02, H7, iPSC4, iPSC5 |
|  |  |  |  | NPC | BG02 |
|  |  |  | Adult | | Lymphoblastoid (C0202) |
| Peric-Hupkes et al. 2010 | Lamin Association Log2(Lamin Associating/Input) | Mouse | Early Embryonic | ESC | Embryonic stem cells (ESC) |
|  |  |  |  | NPC | Neural progenitor cells (NPC) |
|  |  |  | Late Embryonic | AC | Astrocytes (AC) |
|  |  |  |  | MEFF | Embryonic fibroblasts (MEFF) |
| Guelen et al. 2008 | Lamin Association Log2(Lamin Associating/Input) | Human | Late Embryonic | | Embryonic lung fibroblasts (Tig3) |
| Lieberman–Aiden et al. 2009 | Hi-C interaction matric eigenvalues | Human | Adult | | Lymphoblastoid (GM06990) |
| Kalhor et al. 2012 | Hi-C interaction matric eigenvalues | Human | Adult | | Lymphoblastoid (GM12878) |
